# Sleep Habits, Physical Exercise, and Social Media Use and Their Influence on Perceptions of Physical and Mental Health—Case Study at a Higher Education Institution in Portugal

**DOI:** 10.3390/healthcare14030343

**Published:** 2026-01-29

**Authors:** Ana Paula Oliveira, Joana Nobre, Francisco Monteiro, Carlos Rodrigues, Olga Louro, Nelson Valente, Luís Branquinho, Nuno Carrajola, Bruno Morgado

**Affiliations:** 1 Higher School of Health, Portalegre Polytechnic University, 7300-110 Portalegre, Portugal; joana.nobre@ipportalegre.pt (J.N.); luisbranquinho@ipportalegre.pt (L.B.);; 2 CARE—Research Center on Health and Social Sciences, 7300-555 Portalegre, Portugal; 3 Research Center in Sports Sciences, Health Sciences and Human Development (CIDESD), 6201-001 Covilhã, Portugal; 4 Life Quality Research Center (LQRC-CIEQV), 2001-904 Santarém, Portugal; 5 Research Centre of the Higher Institute of Educational Sciences (CI-ISCE), 2620-379 Lisbon, Portugal

**Keywords:** sleep, motor activity, social media, mental health, students

## Abstract

**Background/Objectives:** The transition to higher education is often accompanied by lifestyle changes that may influence sleep habits, physical activity, and social media use, with potential consequences for physical and mental health. **Methods:** A quantitative, cross-sectional, descriptive, and correlational study was conducted using an online questionnaire administered between April and May 2024. The sample included 201 participants (123 students and 78 teaching/non-teaching staff). Data were collected using the Mental Health Inventory-5 (MHI-5), Social Media Addiction Scale (SMAS), Global Physical Activity Questionnaire (GPAQ), and Pittsburgh Sleep Quality Index (PSQI). Descriptive statistics and Spearman correlation analyses were performed. **Results:** Students presented lower mental health scores compared to staff members. Sleep quality indicators, particularly reduced sleep efficiency and increased use of sleep medication, were significantly associated with poorer mental health. Correlations between physical activity, social media use, sleep quality, and mental health were generally weak, suggesting that these domains contribute independently to perceived well-being. Staff members showed slightly higher levels of social media addictive behaviors, while students reported shorter sleep duration and greater emotional variability. **Conclusions:** The findings indicate that students presented lower mental health scores and poorer sleep indicators compared to staff members. Sleep quality—particularly sleep duration, efficiency, and use of sleep medication—showed the most consistent associations with mental health, while physical activity and social media use demonstrated weaker relationships. These results highlight the relevance of targeted sleep-focused interventions within higher education settings, especially for students in low-density regions.

## 1. Introduction

The transition to higher education offers students greater autonomy and new and broader social opportunities, but all of this entails greater individual responsibility and an increased capacity to manage all academic and social tasks. Some students possess this ability, integrate easily, and perform well academically, while others face greater difficulties throughout this process, as significant changes in daily life dynamics combined with high expectations about the university academic experience and high individual and peer expectations may affect initial adjustment [1].

There is reason to believe that physical and mental health may predict psychosocial and academic adjustment and engagement in certain health behaviors among students during their first year at university [2]. Poorer general health is also transversely associated with greater symptoms of anxiety [3]. Increasing numbers of universities worldwide are reporting growing rates of students with mental disorders and, in many cases, an increased demand for internal services that far exceeds available resources [4].

Psychological and lifestyle-related factors may not be determinants of disease, but they contribute significantly to general physical and mental health problems and impair the physical, mental, and social functioning and quality of life of everyone. Therefore, the promotion of mental health is just as important as the promotion of physical health. There is no health without mental health [5].

People’s lifestyles today have changed and are highly influenced by a faster pace of life, greater incentives for consumption, economic changes, and dependence on technology, with impacts on interpersonal relationships and health. About half of the world’s population uses online social networks, spending more than two and a half hours per day on them, with young people aged 16 to 24 representing one-third of these users and spending more time on social networks daily compared with any other age group (3 h 11 min for female, 2 h 46 min for men) [6].

Recent evidence has highlighted the lasting impact of the COVID-19 pandemic on mental health and lifestyle behaviors among young adults. Long-term consequences have included persistent psychological distress, changes in sleep patterns, reduced physical activity, and increased sedentary and screen-based behaviors, even after the acute phase of the pandemic [7]. These effects appear particularly relevant in higher education populations, where academic demands may intensify vulnerability to mental health difficulties. In addition, recent studies have emphasized the central role of sleep habits in young adults, demonstrating significant associations between sleep quality and somatic health indicators, as well as sex-specific differences in these relationships, reinforcing the importance of considering demographic and behavioral factors when examining physical and mental health outcomes in this age group [8,9].

Social networks, understood as internet-based applications that facilitate the exchange and exploration of user-generated content, are an omnipresent part of today’s world, with young adults being the largest users, 60% of whom report using screens before bedtime [10,11]. This recent phenomenon has motivated a growing body of literature on the use of social networks and their effects on sleep quality and mental health among young people. Over the past two decades, social media platform use has grown exponentially among young adults, becoming an integral part of university students’ academic, social, and informational routines. This increase has not only translated into greater connectivity; it has also given rise to problematic or dependent patterns of use [12]. Several studies have reported high prevalence rates of addictive behavior toward social networks among higher education students, with implications at psychological, physical, behavioral, and social levels [13]. Other longitudinal studies suggest that poor sleep quality and frequent sleep disturbances may partly explain the association between excessive social network use and negative impacts on mental health, although there is also current evidence that remains inconclusive regarding the directionality of the relationships between social network use and mental health [10].

Sleep duration is an important factor in its quality, which is defined by four key elements: Sleep Latency (the time required to fall asleep), Awakenings (the number of times a person wakes during the night), Wake After Sleep Onset (the time spent awake after falling asleep), and Sleep Efficiency (the proportion between time asleep and time spent in bed). For healthy young adults and adults with normal sleep, the adequate duration is 7 to 9 h [14]. Sleep duration in university students from 26 low-, middle-, and high-income countries showed prevalence rates of 39.2% for ≤6 h, 46.9% for 7–8 h, and 13.9% for ≥9 h of sleep duration [15]. More intense academic and social pressures, irregular schedules, a variety of entertainment options, unhealthy habits such as smoking and drinking, and other easily accessible leisure activities make students susceptible to sleep disorders and sleep deprivation. Therefore, it can be stated that poor sleep quality is becoming a considerable problem among university students [16]. Restorative sleep is essential for physical and mental health. However, sleep deprivation is a prevalent and urgent public health issue in a fast-paced society where relationships and access to information are strongly influenced by technology and the internet [17].

Sleep deprivation impairs cognitive function and has been associated with poorer academic performance in higher education students. Better sleep quality and knowledge about sleep hygiene have been associated with better academic performance, and better sleep quality and lower levels of chronic sleep deprivation have been linked to better concentration in studies [17].

A recent 2025 study involving around 4100 university students found a significant positive correlation between physical exercise and sleep quality and significant negative correlations with anxiety, depression, and social network addiction. Anxiety and depression also showed a significant negative correlation with sleep quality and a significant positive correlation with social media addiction. The study found that anxiety, depression, and social media addiction play a chain mediating role between physical exercise and sleep quality in university students [18].

Some research suggests a bidirectional relationship between sleep and physical activity in adults and low levels of physical activity with sleep disturbances. A meta-analysis of twenty-nine eligible studies with a total of 141,035 university students showed that moderate-to-vigorous physical activity was associated with better sleep quality and a weak negative association between moderate-to-vigorous physical activity and sleep duration was found [19]. Convincing evidence has demonstrated that physical activity and exercise can prevent common mental disorders such as depression and anxiety disorders and have multiple beneficial effects on the physical and mental health of people with a wide range of mental disorders [20,21].

A meta-analysis by Leow, M. (2023) confirmed in a population of university students that the prevalence of inadequate sleep was 57% and smartphone addiction 39%, demonstrating a correlation index of 0.30 between the two [22].

Sleep quality is one of the health domains most consistently associated with problematic use of electronic devices and social networks [23]. There is strong and consistent evidence that social media addiction significantly impairs sleep quality in adolescents, particularly when use is frequent, emotionally motivated, or occurs at night [14]. Among the proposed mechanisms are exposure to blue light that suppresses melatonin, displacement of sleep time due to nighttime use of social networks, cognitive and emotional activation (e.g., rumination, fear of missing out—FoMO), and mediation by depressive/anxious symptoms [16,24,25,26].

From a conceptual perspective, this study is grounded in a biopsychosocial framework, in which health and well-being result from the interaction between behavioral, psychological, and social factors. Sleep habits, physical activity, and social media use are understood as interrelated lifestyle behaviors that may influence mental health both directly and indirectly. Adequate sleep and regular physical activity are considered protective factors for psychological well-being, whereas excessive or maladaptive use of social media may act as a risk factor by contributing to sleep disturbances, emotional dysregulation, and psychological distress. Within this framework, mental health is viewed as an outcome shaped by the balance between these behaviors, particularly in young adults navigating the demands of higher education.

Despite the growing body of literature addressing sleep habits, physical activity, and social media use, limited research has simultaneously examined these domains within the same analytical framework in higher education communities, particularly in low-density regions. Moreover, most studies focus exclusively on student populations, with scarce attention given to comparisons between students and academic staff. Therefore, the present study contributes to the literature by jointly analyzing sleep habits, physical activity, social media use, and perceived mental health in both students and teaching/non-teaching staff within a single institutional context, offering a more comprehensive perspective on well-being in higher education settings.

The population examined in this study presents distinctive characteristics that justify its investigation. Higher education institutions located in low-density regions face specific structural and social challenges, including reduced access to health services, geographic isolation, limited recreational and physical activity resources, and fewer mental health support structures compared to urban academic settings. These contextual factors may influence lifestyle behaviors and perceptions of well-being in both students and staff. However, empirical evidence focusing on academic communities in low-density regions remains scarce. By addressing this gap, the present study provides insight into how sleep habits, physical activity, and social media use relate to mental health within an underrepresented higher education context.

Therefore, the aim of this study was to examine the associations between sleep habits, physical exercise, and online social media use and their influence on perceived physical and mental health among students and teaching/non-teaching staff at a higher education institution located in a low-density region of Portugal.

## 2. Materials and Methods

### 2.1. Objectives

This study was conducted at a higher education institution in a low-density area of Portugal with the aim of identifying the association between sleep habits, physical exercise, and use of online social networks and their influence on the perception of physical and mental health among students and teaching/non-teaching staff.

### 2.2. Study Design

This quantitative, cross-sectional, descriptive, and correlational study used an online questionnaire on Google^®^ Forms, administered to students and teaching and non-teaching staff at a higher education institution in Portugal. It took place between April and May 2024.

### 2.3. Clinical Assessment Tools

#### 2.3.1. Mental Health Inventory-5 Items (MHI-5)

The Mental Health Inventory-5 (MHI-5) is an abbreviated version of the Mental Health Inventory developed by Veit and Ware, designed to assess psychological well-being and the presence of symptoms of anxiety and depression. It is widely used as a brief measure of mental health in population studies. The Portuguese version was adapted and validated by Pais-Ribeiro, presenting good psychometric qualities, with adequate internal consistency and convergent validity with other mental health indicators.

#### 2.3.2. Social Media Addiction Scale (SMAS)

The Social Media Addiction Scale (SMAS) was originally developed by Jamal Al-Menayes to assess problematic use and dependence on social media. The scale consists of 14 items that measure symptoms related to compulsive use, tolerance, and conflict with other areas of life. The Portuguese version was validated by Leite, Ramires, Lira, and Magano, showing good internal consistency (α > 0.80) and a factorial structure like the original version, confirming its suitability for the Portuguese population.

#### 2.3.3. Global Physical Activity Questionnaire (GPAQ)

The Global Physical Activity Questionnaire (GPAQ) was developed by Armstrong and Bull under the auspices of the World Health Organization to assess physical activity in three domains: work, commuting, and leisure time. The scale is widely used in population surveys and lifestyle research. The Portuguese version was recently validated by Ribeiro et al., showing good reliability indicators (ICC > 0.75) and convergent validity with objective measures of physical activity.

#### 2.3.4. Pittsburgh Sleep Quality Index (PSQI)

The Pittsburgh Sleep Quality Index (PSQI) was created by Buysse et al. to assess sleep quality and patterns over the past month, covering seven components, such as latency, duration, efficiency, and sleep disturbances. The Portuguese version was validated by Del Rio João et al., demonstrating good internal consistency (α = 0.77) and convergent validity with mental health and fatigue indicators.

### 2.4. Data Collection

A non-probabilistic convenience sample was obtained. The questionnaire link was sent by email to the population of 3176 students, 268 teaching staff members, and 179 non-teaching staff members, and a total of 201 valid questionnaires were obtained. The study and its objectives were described at the beginning of the questionnaire, and each participant gave their consent to participate before starting to complete it.

Ethical issues were always respected by the researchers, and the procedures were carried out in accordance with the Declaration of Helsinki. Participants’ anonymity was guaranteed, as no question made it possible to identify any participant. Data confidentiality was ensured, with data stored in a database on the researchers’ personal computers, protected by a security code for access. The study was approved by the Ethics Committee of the institution where the study was conducted (Opinion No. SC/2024/2909 of 22 March 2024).

The ad hoc questions used and developed by the researchers consisted of self-report items specifically designed for this study, such as sex; age; marital status; academic qualifications; area of training/course attended; distance from the school to the usual residence; and perception of general physical health, including vision, hearing, oral health, and emotional balance.

**Figure 1:**
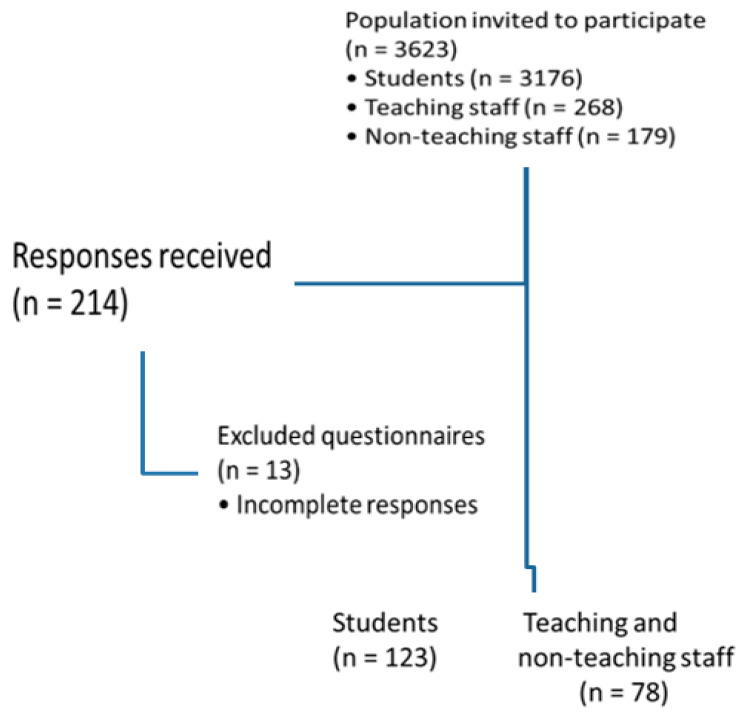
Flowchart of the study design and participant inclusion process.

Before data collection, a pre-test of the questionnaire was administered to ten (10) staff members and ten (10) students who, after completing it, made no suggestions for changes; therefore, the initial version was maintained, Figure 1.

### 2.5. Data Analysis

Data analysis was performed using IBM SPSS Statistics^®^ version 26. Descriptive statistics were used to characterize the sample and summarize the main study variables. Categorical variables were presented as absolute and relative frequencies, while continuous variables were described using means and standard deviations. Prior to inferential analyses, the distribution of all variables from the MHI-5, SMAS, GPAQ, and PSQI scales was assessed for normality using the Shapiro–Wilk test, given its suitability for small to moderate sample sizes. Most variables did not meet the assumption of normality (*p* ≤ 0.05). Consequently, nonparametric statistical procedures were adopted. Spearman’s rank correlation coefficient (ρ) was used to examine associations between sleep quality indicators, physical activity variables, social media use, and mental health scores. Correlation strength was interpreted according to conventional criteria (weak: <0.30; moderate: 0.30–0.59; strong: ≥0.60). A significance level of *p* < 0.05 was considered statistically significant for all analyses. Regarding sample size adequacy, the final sample of 201 participants was considered sufficient for correlational analyses, allowing the detection of small-to-moderate effect sizes with adequate statistical power in accordance with recommendations for nonparametric correlation studies.

## 3. Results

The sample included 123 students and 78 staff members (teaching and non-teaching). The most representative group of students is female, aged up to 25 years, single but in a romantic relationship; their family residence is located more than 200 km from the school, and during the academic period they live in a rented single room.

The most representative group of staff members is female, aged between 41 and 50 years, married/in a de facto union, holds a bachelor’s degree, and resides in their own home at a distance of 3 to 15 km from the school.

Regarding the perception of general physical health, both groups consider it to be good. With respect to the perception of balance and mental health, students consider it to be fair, while staff members consider it to be good (Table 1).

Regarding students’ mental health in the last month, the most representative group reported: feeling very nervous almost all the time; feeling calm and peaceful some of the time; feeling sad and down some of the time; feeling sad and down to the extent that nothing could cheer them up infrequently; however, most of the time they felt like a happy person. Regarding staff members’ mental health in the last month, the most representative group reported: feeling nervous some of the time; feeling calm and peaceful most of the time; feeling sad and down almost never; never feeling so sad and down that nothing could cheer them up; and feeling like a happy person most of the time. As can be observed, students’ mental health index is lower across all dimensions (Table 2).

Table 3 shows the mean scores obtained in the two dimensions of the Social Media Addiction Scale (SMAS) and the total score, comparing students and staff. Overall, staff exhibited slightly higher values across all evaluated dimensions. In the ‘Compulsive’ factor—which reflects automatic and difficult-to-control social media behavior, staff reported higher mean scores (M = 19.27) than students (M = 16.55). Similarly, in the ‘Social’ factor—related to social engagement and the need for online interaction—staff also presented higher values (M = 7.08) compared to students (M = 6.24). Regarding the total SMAS score, staff showed a higher overall level of addictive social media behavior (M = 31.99) than students (M = 29.96), although the differences between the groups were small.

Table 4 presents the mean scores reported by students and staff regarding the different indicators of the Global Physical Activity Questionnaire (GPAQ). Overall, both groups showed similar profiles, with small variations across specific activity dimensions. Students reported a slightly higher number of days per week engaging in vigorous activity (M = 0.81) compared to staff (M = 0.73). However, staff indicated a marginally higher daily duration of vigorous activity (M = 0.59 h/day) than students (M = 0.56 h/day). With regard to moderate intensity activity, students presented both a higher weekly frequency (M = 1.65 days) and longer daily duration (M = 0.95 h/day) than staff (M = 1.33 days; M = 0.84 h/day). Concerning sedentary behavior, staff reported a greater number of sedentary hours per day (M = 3.85 h/day) compared to students (M = 2.90 h/day), suggesting a potentially less active daily routine during work or leisure time.

Table 5 presents the mean scores obtained by students and staff across the different PSQI components. Overall, small differences were observed between the groups, although distinct patterns emerged in each sleep dimension. Staff reported a higher daily sedentary time (M = 3.85 h/day) compared to students (M = 2.90 h/day), suggesting a more sedentary daily routine. Students presented higher sleep duration component scores (M = 1.05) compared to staff (M = 0.83), indicating shorter sleep duration and poorer sleep quality among students, given that higher PSQI scores represent worse sleep outcomes. Regarding sleep efficiency, both groups demonstrated low value, although staff exhibited slightly better efficiency (M = 0.57) than students (M = 0.45). In terms of sleep disturbances, the two groups presented very similar results (students: M = 1.09; staff: M = 1.10), suggesting that both populations experience nighttime disruptions to a comparable extent. Finally, students reported more frequent use of sleep medication (M = 0.55) than staff (M = 0.30), although both values remained relatively low.

The Shapiro–Wilk test was applied to all variables from the SMAS, GPAQ, PSQI, and MHI-5 scales to assess the assumption of normality in the data distributions. This test is particularly suitable for small to medium sample sizes (*n* < 2000) and is sensitive to deviations from normality. The decision criterion adopted was: *p* > 0.05 indicates that the variable follows a normal distribution, while *p* ≤ 0.05 indicates a non-normal distribution. The analysis showed that only the variables ‘SMAS_factor1_compulsive’ and ‘SMAS_total score’ demonstrated a normal distribution (*p* > 0.05). All remaining variables, including the GPAQ dimensions, PSQI components, and the MHI-5 score, exhibited non-normal distributions (*p* ≤ 0.05). Therefore, the Spearman correlation coefficient (ρ) was selected, as it is appropriate for nonparametric data and does not assume linearity or homoscedasticity.

Spearman’s correlation results demonstrated consistent relationships within each scale and between some mental health and sleep domains. A moderate positive correlation was found between the SMAS factors (ρ = 0.561; *p* < 0.001), indicating internal coherence of the scale. Regarding physical activity variables (GPAQ), strong correlations were observed between the number of days and the daily time spent in vigorous (ρ = 0.921; *p* < 0.001) and moderate (ρ = 0.796; *p* < 0.001) physical activity, highlighting consistency between the frequency and duration of physical exercise. Within the PSQI, sleep duration and sleep efficiency were positively correlated (ρ = 0.527; *p* < 0.001), whereas greater use of sleep medication and lower sleep efficiency were negatively correlated with mental well-being (MHI-5), suggesting that poorer sleep patterns are associated with worse mental health outcomes. Correlations between the different scales (SMAS, GPAQ, PSQI, and MHI-5) were generally weak, indicating that each instrument assesses distinct domains—digital dependence, physical activity, sleep quality, and mental health—with independent contributions to overall well-being.

All reported Spearman correlation coefficients are presented together with corresponding *p*-values, with statistical significance defined as *p* < 0.05.

## 4. Discussion

The findings of this study provide valuable insights into the relationships between sleep habits, physical exercise, social media use, and perceived physical and mental health within an academic community. Overall, both students and staff reported generally positive perceptions of their health, though clear differences emerged between the groups.

Students were predominantly young, female, single, and living away from their family homes characteristics that align with typical demographic profiles in higher education [1,27]. This life stage often involves considerable adjustment challenges, as students navigate academic pressures, social expectations, and independence. Prior research indicates that the transition to university life can exacerbate vulnerability to stress and anxiety, especially when compounded by irregular sleep patterns and limited social support [2]. Consistent with these findings, many students in this study reported frequent feelings of nervousness and sadness, although they also expressed moments of calmness and happiness, suggesting emotional fluctuation characteristic of this developmental phase.

Among faculty and staff, perceptions of physical and mental health were more positive and stable, with lower frequencies of reported negative emotions. This may reflect greater emotional maturity, professional stability, and social support factors that have been widely recognized as protective for mental health [5]. Moreover, a higher proportion of married or cohabiting individuals among staff could explain the higher well-being scores, as social connectedness has been shown to buffer psychological distress [4]

Perceptions of physical health were generally good or fair across groups, particularly in auditory and oral health. However, students demonstrated greater variability in emotional balance and mental health self-assessment. This aligns with previous studies showing a progressive decline in psychological well-being throughout university years, often related to academic stress, social isolation, and intensive use of social networks [13,28]

The role of social media use in this dynamic is significant. Excessive or late night use has been associated with sleep disturbances and higher rates of depressive and anxiety symptoms [17,29]. Although this study did not quantify social media use directly, the literature consistently identifies young adults as particularly susceptible to problematic use patterns [13]. Poor sleep quality has been one of the most reliable correlates of lower mental health scores [23,24], suggesting that nighttime digital engagement and academic demands may jointly undermine students’ rest and well-being.

Regular physical activity, although not analyzed in detail here, is known to protect against both physical and mental health deterioration. Evidence demonstrates that students engaging in consistent exercise report fewer depressive symptoms, better sleep, and greater vitality [1,5]. Therefore, promoting physical activity and health literacy within academic settings remains a priority.

These results emphasize the need for integrated health promotion strategies within higher education institutions. Interventions combining sleep hygiene education, responsible digital engagement, and encouragement of physical activity can substantially enhance well-being across the academic community [4,5].

Furthermore, the correlational findings from this study reinforce the multidimensional nature of well-being in academic contexts. The generally weak correlations observed between digital media use (SMAS), physical activity (GPAQ), sleep quality (PSQI), and mental health (MHI-5) suggest that each domain contributes independently to overall health. This pattern supports contemporary evidence indicating that no single health behavior sufficiently explains psychological functioning; rather, well-being results from the combined balance of multiple lifestyle factors [2]. Therefore, interventions focused solely on sleep, physical activity, or technology use may have limited impact unless incorporated into comprehensive, multisectoral strategies.

Despite weak cross-scale correlations, notable dynamics emerged within specific domains. Strong associations between frequency and duration of vigorous physical activity indicate a subgroup with structured exercise behaviors, which is meaningful because consistent physical activity is widely identified as a protective factor against poor sleep and psychological distress [20,30]. Evidence shows that regular exercise reduces rumination and depressive symptoms and supports better sleep regulation, Yin et al., 2025, highlighting the importance of encouraging active lifestyles within university communities [30].

Sleep quality showed significant associations with mental health outcomes, particularly regarding medication use and reduced sleep efficiency. This aligns with studies demonstrating that sleep disturbances are among the most reliable correlations of anxiety and depression in young adults [6,24]. Importantly, problematic sleep may operate as a mechanism linking other behaviors to well-being. Recent research shows that late-night or excessive digital media use contributes to poor sleep, which in turn increases vulnerability to emotional difficulties [31]. This suggests that although direct associations between digital dependence and mental health were weak in this sample, indirect or mediated pathways likely exist but were not captured by simple correlations.

These insights point toward a heterogeneity of risk profiles within academic communities. For example, individuals presenting poor sleep combined with high social media engagement and low physical activity may be at heightened psychological risk, while those maintaining healthy habits across domains appear more resilient. Moderators such as gender, living situation, academic stress, and social support are likely to shape these relationships [2], and future research may benefit from subgroup analyses or longitudinal designs that identify trajectories of risk and protection across university years.

Methodologically, the findings emphasize the limitations inherent to self-report measures and cross-sectional research, which constrain causal interpretation. Future research should consider objective activity/sleep monitoring (e.g., actigraphy, digital tracking) and temporal analyses capable of modeling, mediating and moderating effects across behavioral systems [31].

Taken together, the results of this study underscore the need for holistic health promotion planning in higher education settings. Beyond psychological services, universities should implement coordinated initiatives integrating physical activity promotion, sleep hygiene education, and responsible digital engagement. Such integrated strategies are most likely to enhance students’ long-term well-being and academic success, particularly in low-density regions where access to mental health resources may be limited. The observed independence of health domains highlights an important practical implication: the most effective interventions will be those that simultaneously target multiple lifestyle behaviors, supporting students and staff in developing sustainable routines that protect both physical and mental health.

### Limitations

This study has several limitations that should be acknowledged. First, the use of a non-probabilistic convenience sample limits the generalizability of the findings. Second, the cross-sectional design does not allow causal inferences between lifestyle behaviors and mental health outcomes. Third, all data were collected through self-report instruments, which may be subject to recall bias and social desirability bias. Additionally, the analyses were based on bivariate correlations, which restrict the ability to examine the combined or interactive effects of multiple variables. The cross-sectional design of this study influences the interpretation of the findings. While it allows the identification of associations between sleep habits, physical activity, social media use, and mental health at a specific point in time, it does not permit the assessment of temporal relationships or causal effects. Therefore, the observed associations should not be interpreted as evidence of directionality or causation. Longitudinal studies are required to better understand the dynamic and potentially bidirectional relationships among these variables. Future research would benefit from the use of multivariate statistical models and stratified analyses by group to further explore these relationships. Furthermore, no a priori power analysis was conducted, which represents an additional limitation. Although the total sample size (*n* = 201) was considered adequate for descriptive and correlational analyses, the reduced size of specific subgroups, particularly staff members (*n* = 78), may have limited the statistical power to detect smaller effects. Therefore, the findings should be interpreted with caution, especially regarding subgroup-level interpretations. Future studies with larger and more balanced samples are recommended to support more robust statistical analyses. Despite these limitations, the present study provides relevant insights into the multidimensional nature of well-being in higher education contexts, particularly in low-density regions.

## 5. Conclusions

This study explored the interplay between sleep habits, physical exercise, social media use, and perceptions of physical and mental health in a higher education community situated in a low-density region of Portugal.

Findings revealed distinct patterns between students and staff, with students exhibiting greater variability in mental health indicators and emotional well-being. Among students, frequent nervousness and sadness coexisted with moderate levels of happiness, reflecting the emotional instability often associated with the transition to university life. In contrast, staff members demonstrated more stable and positive perceptions of both physical and mental health, likely due to emotional maturity, professional stability, and stronger social support networks.

The transition to higher education represents a critical stage for developing healthy lifestyle behaviors. Risk factors such as sleep deprivation, sedentary habits, and problematic social media use are well documented threats to psychological and physical well-being. The present findings highlight the importance of fostering an institutional culture that values mental health and balance, encouraging healthy sleep routines, regular physical activity, and mindful digital engagement. Promoting health within academic institutions should therefore adopt a holistic approach, integrating physical, psychological, and social dimensions. Multidisciplinary interventions that combine health education, digital literacy, and psychological support can enhance both quality of life and academic performance. Limitations of the study include its cross-sectional nature and reliance on self-reported data, which restrict causal interpretation. Nevertheless, the results offer valuable insights into the determinants of health in Portuguese higher education and provide an evidence base for institutional strategies aimed at prevention and well-being promotion. Future research should employ larger, representative samples and longitudinal designs to examine changes in sleep, exercise, and social media behaviors over time. Incorporating objective measures such as actigraphy, digital usage monitoring, and physiological stress indicators would strengthen the validity of findings and deepen understanding of underlying mechanisms.

## Figures and Tables

**Table 1:** Sociodemographic characterization of the sample.

Variable	Category	Students (*n*)	Students (%)	Staff (*n*)	Staff (%)
Sex	Male	26	21.1	26	33.3
	Female	95	77.2	52	66.7
	Prefer not to answer	2	1.6	0	0
Age	Up to 25 years	106	86.2	2	2.6
	25–30 years	7	5.7	1	1.3
	31–40 years	5	4.1	7	9
	41–50 years	5	4.1	35	44.9
	51–60 years	0	0	25	32.1
	Over 60 years	0	0	8	10.3
Marital status	Single, without romantic relationship	56	45.5	6	7.7
	Single, with romantic relationship	60	48.8	8	10.3
	Married/de facto union	5	4.1	52	66.7
	Separated/divorced/widowed	2	1.6	12	15.4
Completed academic level	Up to 9th grade	0	0	2	2.6
	12th grade	119	96.7	15	19.2
	Bachelor’s degree	4	3.3	25	32.1
	Master’s degree	0	0	14	17.9
	Doctorate	0	0	22	28.2
Distance from institution to residence	Up to 2 km	19	15.4	18	23.1
	3–15 km	29	23.6	38	48.7
	16–50 km	14	11.4	10	12.8
	51–200 km	30	24.4	7	9
	More than 200 km	31	25.2	5	6.4
Type of residence (work/study days)	Rented house	21	17.1	9	11.5
	Own house	30	24.4	59	75.6
	Rented single room	38	30.9	5	6.4
	Rented shared room	4	3.3	1	1.3
	Family home	8	6.5	3	3.8
	Institutional residence	22	17.9	1	1.3
Perception of general physical health	Very good	13	10.5	6	7.7
	Good	60	48.8	43	55.4
	Fair	45	36.6	26	33.3
	Poor	3	2.4	3	3.8
	Very poor	2	1.6	0	0
Perception of balance and mental health	Very good	10	8.1	17	21.8
	Good	39	31.7	39	50
	Fair	50	40.6	18	23
	Poor	20	16.2	4	5.1
	Very poor	4	4	0	0

**Table 2:** Mental health index of students and staff.

Item	Response Category	Students (*n*)	Students (%)	Staff (*n*)	Staff (%)
During the past month, how much of the time did you feel very nervous?	All of the time	7	5.6	1	1.2
	Most of the time	36	29.3	5	6.4
	A good bit of the time	35	28.4	11	14.2
	Some of the time	35	28.4	30	38.4
	A little of the time	10	8.1	23	29.5
	None of the time	0	0.0	8	10.3
	MHI-5 score (item)		3.08		4.19
During the past month, how much of the time did you feel calm and peaceful?	All of the time	2	1.6	6	7.6
	Most of the time	8	6.5	17	21.8
	A good bit of the time	26	21.1	27	34.7
	Some of the time	52	42.3	18	23.0
	A little of the time	33	26.8	10	12.9
	None of the time	2	1.6	0	0.0
	MHI-5 score (item)		3.04		3.88
During the past month, how much of the time did you feel downhearted and depressed?	All of the time	2	1.6	1	1.2
	Most of the time	12	9.7	2	2.4
	A good bit of the time	29	23.6	6	7.5
	Some of the time	52	42.7	21	27.0
	A little of the time	25	20.3	42	55.1
	None of the time	3	2.4	6	7.5
	MHI-5 score (item)		3.77		4.51
During the past month, how much of the time did you feel so down that nothing could cheer you up?	All of the time	2	1.6	1	1.8
	Very often	12	9.7	4	5.1
	Often	20	16.3	4	5.1
	Sometimes	46	37.4	18	23.0
	Rarely	26	21.1	15	19.2
	Never	17	13.8	36	46.2
	MHI-5 score (item)		4.08		4.44
During the past month, how much of the time did you feel happy?	All of the time	9	7.3	6	7.5
	Most of the time	26	21.1	28	36.0
	A good bit of the time	62	50.4	30	35.8
	Some of the time	24	19.5	13	16.7
	A little of the time	2	1.6	1	1.2
	MHI-5 score (item)		3.91		4.14
Total Mental Health Index (MHI-5)			17.89		21.65

**Table 3:** Social Media Addiction Scale (SMAS).

Scale	Students (Mean ± SD)	Staff (Mean ± SD)
Factor 1—Compulsions	16.55 ± 4.57	19.27 ± 4.58
Factor 2—Social	6.24 ± 2.58	7.08 ± 2.61
Total Score (SMAS)	29.96 ± 7.15	31.99 ± 6.68

**Table 4:** Global Physical Activity Questionnaire (GPAQ).

Indicator	Students (Mean ± SD)	Staff (Mean ± SD)
Days of vigorous physical activity (per week)	0.81 ± 1.46	0.73 ± 1.33
Days of moderate physical activity (per week)	1.65 ± 2.03	1.33 ± 1.77
Daily time of vigorous physical activity (h/day)	0.56 ± 1.11	0.59 ± 1.18
Daily time of moderate physical activity (h/day)	0.95 ± 1.35	0.84 ± 1.18
Daily sedentary time (h/Day)	2.90 ± 1.84	3.85 ± 2.21

**Table 5:** Pittsburgh Sleep Quality Index (PSQI).

Dimension	Students (Mean ± SD)	Staff (Mean ± SD)
Sleep latency	1.06 ± 0.93	1.41 ± 0.98
Sleep duration	1.05 ± 0.88	0.83 ± 0.79
Sleep efficiency	0.45 ± 0.70	0.57 ± 0.77
Sleep disturbances	1.09 ± 0.55	1.10 ± 0.52
Use of sleep medication	0.55 ± 0.99	0.30 ± 0.78

## Data Availability

The original contributions presented in this study are included in the article. Further inquiries can be directed to the corresponding author.
